# The bioequivalence study design recommendations for immediate-release solid oral dosage forms in the international pharmaceutical regulators programme participating regulators and organisations: differences and commonalities

**DOI:** 10.3389/jpps.2024.12398

**Published:** 2024-03-21

**Authors:** Eduardo Agostinho Freitas Fernandes, Joy van Oudtshoorn, Andrew Tam, Liliana Carolina Arévalo González, Erwin Guzmán Aurela, Henrike Potthast, Katalina Mettke, Ryosuke Kuribayashi, Kohei Shimojo, Miho Kasuga, Lázaro Morales, Zulema Rodríguez, Ben Jones, Choongyul Ahn, Eunju Yun, So Hee Kim, Clare Rodrigues, Toh Tiong, Christopher Crane, Chantal Walther, Matthias S. Roost, Tzu-Ling Chen, Li-feng Hsu, April C. Braddy, Alfredo García-Arieta, Ivana Abalos, Milly Divinsky, Abdulaziz Alsuwyeh, Bader Alzenaidy, Adel Alharf

**Affiliations:** ^1^ Agência Nacional de Vigilância Sanitária, Brasília, Brazil; ^2^ South African Health Products Regulatory Authority, Pretoria, South Africa; ^3^ Health Canada, Ottawa, ON, Canada; ^4^ Instituto Nacional de Vigilancia de Medicamentos y Alimentos, Bogota, Colombia; ^5^ Federal Institute for Drugs and Medical Devices, Bonn, Germany; ^6^ Ministry of Health, Labour and Welfare/Pharmaceuticals and Medical Devices Agency, Tokyo, Japan; ^7^ Comisión Federal para la Protección contra Riesgos Sanitarios, Ciudad de Mexico, México; ^8^ Medsafe, Wellington, New Zealand; ^9^ Ministry of Food and Drug Safety, Cheongju-si, Republic of Korea; ^10^ Health Sciences Authority, Singapore, Singapore; ^11^ Therapeutic Goods Administration, Canberra, NSW, Australia; ^12^ Swissmedic, Bern, Switzerland; ^13^ Taiwan Food and Drug Administration, Taipei, Taiwan; ^14^ Center for Drug Evaluation, Taipei, Taiwan; ^15^ Food and Drug Administration, Silver Spring, MD, United States; ^16^ WHO-Observer, Geneva, Switzerland; ^17^ Agencia Española de Medicamentos y Productos Sanitarios, Madrid, Spain; ^18^ Administración Nacional de Medicamentos, Alimentos y Tecnología, Buenos Aires, Argentina; ^19^ Center for Pharmaceutical and Enforcement Division, Jerusalem, Israel; ^20^ Saudi Food and Drug Authority, Riyadh, Saudi Arabia

**Keywords:** bioequivalence, IPRP, immediate release, study design, ICH

## Abstract

Bioequivalence (BE) studies are considered the standard for demonstrating that the performance of a generic drug product in the human body is sufficiently similar to that of its comparator product. The objective of this article is to describe the recommendations from participating Bioequivalence Working Group for Generics (BEWGG) members of the International Pharmaceutical Regulators Programme (IPRP) regarding the conduct and acceptance criteria for BE studies of immediate release solid oral dosage forms. A survey was conducted among BEWGG members regarding their BE recommendations and requirements related to study subjects, study design, sample size, single or multiple dose administration, study conditions (fasting or fed), analyte to be measured, selection of product strength, drug content, handling of endogenous substances, BE acceptance criteria, and additional design aspects. All members prefer conducting single dose cross-over designed studies in healthy subjects with a minimum of 12 subjects and utilizing the parent drug data to assess BE. However, differences emerged among the members when the drug’s pharmacokinetics and pharmacodynamics become more complex, such that the study design (e.g., fasting *versus* fed conditions) and BE acceptance criteria (e.g., highly variable drugs, narrow therapeutic index drugs) may be affected. The survey results and discussions were shared with the ICH M13 Expert Working Group (EWG) and played an important role in identifying and analyzing gaps during the harmonization process. The draft ICH M13A guideline developed by the M13 EWG was endorsed by ICH on 20 December 2022, under *Step 2*.

## Introduction

The increasing availability of high-quality generic medicinal products plays an important role in promoting access to medicines worldwide and helping to address rising health care costs. However, this has resulted in significant pressures on regulatory authorities tasked with reviewing and approving these products.

In order to address the challenges posed by increasing workload, globalisation and complexity of scientific issues, the Bioequivalence Working Group for Generics (BEWGG) of the International Pharmaceutical Regulators Programme (IPRP), previously known as the Bioequivalence Working Group (BEWG) of the International Generic Drug Regulators Programme (IGDRP), was created to promote collaboration, regulatory convergence and mutual reliance on respective BE assessments in the long run [1, 2].

The BEWGG is composed of the following regulators/agencies: ANMAT, Argentina; ANVISA, Brazil; COFEPRIS, Mexico; EC, Europe; Health Canada, Canada; HSA, Singapore; INVIMA, Colombia; Medsafe, New Zealand; SAHPRA, South Africa; MFDS, Republic of Korea; CPED, Israel; MHLW/PMDA, Japan; Swissmedic, Switzerland; TFDA, Chinese Taipei; TGA, Australia; FDA, United States; SFDA, Saudi Arabia as well as an observer, WHO.

A BE study is to determine whether there is a relevant formulation effect that significantly influences the bioavailability of the test product in comparison to the comparator product. BE implies that the test product can be expected to be therapeutically equivalent to the comparator product when administered to patients under the specified conditions of use. Different regulatory requirements for the design and analysis of BE studies around the World could result in an additional burden for the development of affordable generic medicines and non-optimal requirements in some countries.

The purpose of this work is to summarise the regulatory requirements for the design and analysis of BE studies of oral immediate-release (IR) products. The information and discussions generated from this survey have proven to be useful regarding the development of the ICH M13A guideline [3] and may continue to be useful to industry and regulatory agencies in understanding the scientific rationale and regulatory considerations pertaining to the standardization of BE study design and acceptance criteria for IR solid oral dosage forms.

## Materials and methods

The IPRP BEWGG conducted a survey to collect recommendations from each participant regarding the design and analysis of BE studies for IR solid oral dosage forms. This information was obtained from the participating members respective regulatory guidance documents and policies [4–28].

## Results

The following sections discuss the key findings of the survey in relation to aspects of study design.

### Cross-over and parallel study designs

For all survey participants, the most common study design is a two-period cross-over study. This type of study design is where each subject is administered both the test and comparator formulations, with each subject acting as their own control. The advantage of using a two-period cross-over study design is that, with the same number of observations, the residual error, composed mostly of the intra-subject variability, is always lower than the residual error in a parallel design, which encompasses inter-subject variability, thereby allowing the enrollment of a smaller number of subjects.

There are other points to consider when selecting certain study designs. For example, the replicate cross-over study design may be selected when there is high intra-subject variability. In these instances, study formulations are administered to the same subject more than once, requiring fewer subjects due to the increased number of observations. This design also allows for the determination of the intra-subject variability of the replicated formulation. On the other hand, a cross-over study design without a drug-free period (i.e., active washout) between formulations may be appropriate for studies conducted at steady state in patients where it would be unethical to discontinue treatment during a washout period (passive washout). Instead of a drug-free washout period, the study drugs are administered for a long enough duration prior to sampling, to allow for elimination of the previously administered formulation.

Although infrequent in the context of generic medicinal products, the consideration of more than two formulations for comparison (e.g., in pilot studies to select the formulations for the pivotal study) or the comparison of formulations under varying conditions (e.g., to investigate the food effect under different conditions: fasting *vs*. low fat fed *vs*. high fat fed), may need the consideration of additional periods and sequences. Parallel designed studies may be useful when studying drugs with long elimination half-lives or some depot formulations to avoid the extended washout period necessary in the case of cross-over designs, which would increase the probability of dropouts. However, any imbalance between groups may cause differences that are not related to the treatments under comparison (e.g., metabolic status, sex, body weight or any other demographic characteristic that may affect the pharmacokinetics of the drug under investigation and which may be unknown). Consequently, a two-period cross-over design is generally recommended even in the case of large washout periods.

### Single dose and multiple dose studies

A BE study may be conducted by administering the same molar dose of each product, preferably as a single unit dosage form. For all survey participants a single dose study is generally preferred as it has a greater sensitivity to detect differences in the rate and extent of absorption between IR formulations.

When the highest strength is not tolerated in healthy volunteers, conducting a study in patients with the highest strength is acceptable to all and preferred in Australia and the United States. Argentina, Brazil, Canada, Colombia, the EU, New Zealand, Republic of Korea, Saudi Arabia, South Africa, Chinese Taipei, and the WHO instead prefer a study using a tolerated intermediate strength in healthy subjects rather than a study in patients. Israel, Japan, Mexico, Singapore, and Switzerland assess the issue on a case-by-case basis. A waiver from conducting BE studies for any additional strengths would be conditional on the recommendations in each jurisdiction [29].

When the test product cannot be administered to healthy volunteers at any therapeutic dose (or at the recommended dose) due to safety concerns, multiple dose studies in patients are considered appropriate. This is particularly relevant when the patient’s treatment cannot be interrupted, as would be required for a single dose study with a washout period.

In the case of drugs that are able to induce their own metabolism, such as carbamazepine, enrolling patients may be necessary in the BE study, since they represent the most sensitive population and regularly use the drug [30]. However, none of the IPRP members recommend conducting a BE study in patients because any sensitivity gained by using patients is negated by the multiple-dose study design.

### Sample size

Another factor considered in the survey is the number of subjects that would participate in the BE study. The sample size should provide enough power (at least ≥80%) to allow for the demonstration of BE between the tested products based on the primary pharmacokinetic parameters (i.e., AUC and C_max_). In general terms, a larger sample size will yield greater power and a more precise outcome.

Brazil, Japan, Mexico, Saudi Arabia, and the United States, recommend a calculation to ensure a power greater than 80%. While Argentina, Australia, Canada, Colombia, the EU, Israel, New Zealand, Republic of Korea, Singapore, South Africa, Switzerland, Chinese Taipei, and the WHO, do not have such a recommendation in the guideline. For ethical reasons, the studies should be sufficiently powered to be able to demonstrate their objectives before the study is started, but this topic is not addressed in some guidelines. Despite the lack of power greater than 80%, all members recommend that all calculations are based on maintaining the overall Type I error at 5% by using 90% confidence intervals.

Upon study completion, Brazil requires that the post-study power be at least 80% to validate the study conclusion. If the calculated post-study power, based on the observed test/comparator point estimate and intra-subject variability is lower than 80%, then a test to detect the differences between the marginal distributions of the test and comparator formulations is required (e.g., Pitman-Morgan adjusted F-test). Insufficient post-study power could be due to higher variability of the test product compared to the comparator, raising concerns about the conclusion of BE. In the case where the variability of test product is considered not statistically different to that of comparator, the study would be accepted. Conversely, if this variability is considered statistically different, the test is considered failed, and the study would be rejected [31].

Except for Japan, all survey participants would accept a pilot study initiated without a sample size calculation, if it has been conducted with at least 12 participating subjects. Japan accepts only pivotal study data.

In the case where a subject is withdrawn from the study before the first dosing period, and it is pre-specified in the study protocol, Argentina, Australia, Brazil, Canada, Colombia, the EU, Japan, Mexico, New Zealand, Republic of Korea, Singapore, Saudi Arabia, South Africa, Switzerland, Chinese Taipei, the United States, and the WHO, would allow the recruitment of additional subjects to replace the non-dosed subject(s), while Israel does not address this possibility.

When the drop-out rate is higher than expected after beginning the study, and it is pre-specified in the study protocol, Argentina, Brazil, Canada, the EU, New Zealand, Singapore, South Africa, Switzerland, Chinese Taipei, the United States, and the WHO allow the inclusion of new subjects during the study, but only before sample bioanalysis.

All regulators recommend that all subjects that have completed a study as per the study protocol be included in the statistical analysis, even if there are more subjects than necessary to conclude BE according to the study power.

In circumstances where information regarding the proposed estimate of the intra-subject variance is absent or lacking, conducting a BE study in stages to initially determine the intra-subject variance is possible. Argentina, Australia, Canada, Colombia, the EU, Japan, Mexico, New Zealand, Singapore, Saudi Arabia, South Africa, Switzerland, the United States, and the WHO, allow the use of the observed intra-subject variance from the first stage to determine the final sample size. For instance, a Group Sequential Design and Adaptive Design are two types of designs that allow data to be collected in stages. In both types of designs, the overall Type I error should be maintained at 5% and the algorithm should be defined *a priori* in the protocol. Typically, for the simplest two-stage design with a group sequential design and stages of equal size, the 94.12% (or 95%) confidence interval for the ratio test/comparator of the primary pharmacokinetic parameters must lie within 80.00–125.00%. These approaches are applicable to both cross-over and parallel study designs. Brazil, Israel, Japan, Republic of Korea, and Chinese Taipei have not addressed the possibility of conducting a BE study in stages.

### Study condition (fasting or fed)

When conducting BE studies, it is important to consider whether the study dose should be given under fasting, fed or both conditions. The BEWGG participants take into account several factors to determine the relevant condition(s) before conducting a single or multiple studies, including dosing administration stated in the product labelling, the drug pharmacokinetics (e.g., linear vs. non-linear), the possible effect of food on drug pharmacokinetics, participant safety, drug solubility, and the pharmaceutical dosage form. The reasons for deciding which studies should be conducted under fasting and/or fed conditions to demonstrate BE varies among the survey participants.

With the exception of the Japan and the United States, if a drug product label specifies administration under fasting conditions only or fed conditions only, then a BE study would be required under the indicated condition for a majority of IPRP participants (Table 1). The US generally recommends the conduct of both fasting and fed studies unless there are safety concerns for participants in the non-recommended state. In Japan, a BE study should be conducted in a fasted state, even if the comparator product is recommended to be taken in fed state, provided that the study is safe for participants and the concentrations are measurable.

**TABLE 1:** Recommended BE Studies according to the content of package insert of comparator product.

	To be administered only in fasting condition	To be administered only in fed condition	To be administered in both fasting and/or fed condition
Argentina	Fasting	Fed	Fasting
Australia	Fasting	Fed	Fasting
Brazil	Fasting	Fed	Fasting^a^
Canada	Fasting	Fed	Fasting
Chinese Taipei	Fasting	Fed	Fasting
Colombia	Fasting	Fed	Fasting
EU	Fasting	Fed	Fasting^a^
Israel	Fasting	Fed	Fasting
Japan	Fasting	Fasting^b^	Fasting
Mexico	Fasting	Fed	Fasting or Fed
New Zealand	Fasting	Fed	Fasting
Republic of Korea	Fasting	Fed	Fasting
Saudi Arabia	Fasting	Fed	Fasting
Singapore	Fasting	Fed	Fasting
South Africa	Fasting	Fed	Fasting
Switzerland	Fasting	Fed	Fasting
United States	Fasting + Fed^c^	Fasting + Fed^d^	Fasting + Fed
WHO	Fasting	Fed	Fasting

^a^ Only when bioavailability is not affected by food or bioavailability is affected by food, but it is not clinically relevant.

^b^ When the BA evaluations from the aspects of bioassay (e.g., drug concentration is very low) are difficult or high incidence of severe adverse events is anticipated in fasting condition, study in fed condition is accepted. In this situation, the low-fat meal is given.

^c^ A fed study is not recommended when the packaging insert (RLD) states the product should be taken on an empty stomach due to safety concerns for subjects.

^d^ A fasting study is not recommended when there are safety concerns for subjects.

When the comparator product can be taken either in a fasted state or in a fed state, most of the members recommend only a fasted state study. Mexico accepts a fasted or a fed study. In the United States, both fasted and fed studies are recommended. Brazil would also require both fasting and fed studies if the bioavailability of a drug product is known to be affected by food and there is uncertainty around whether the food effect is clinically significant. For instance, the package insert for buspirone indicates that the product can be taken with or without food; however, food significantly increases the bioavailability of buspirone, yet the clinical significance of the increase is unknown. Similarly, although not stated in the EMA Guideline on the investigation of BE, some product-specific BE guidances in the EU requires both fasted and fed studies when the comparator product can be taken either in fasted or fed state, but the patient must consistently choose one method of administration. The rationale behind this requirement is that the differences in bioavailability between fasted and fed state are clinically relevant, and BE must be confirmed under both conditions [32–35].

From some survey participants, it is also important to consider the pharmacokinetics of the comparator product when determining the studies to be conducted. For comparator products with non-linear pharmacokinetics, Israel would require studies conducted under both fasting and fed conditions if the comparator can be administered in either state. In Canada, for drugs with non-linear pharmacokinetics within the single unit dose range of approved strengths due to limited solubility of the medicinal ingredient and resulting in less than proportional increases in AUC with increasing dose, the comparative bioavailability studies should be conducted on at least the lowest strength (single dose unit) in the fasted state and the highest strength in both the fasted and fed states.

In Canada, both fasted and fed studies are required for critical dose drugs (i.e., drugs where comparatively small differences in dose or concentration result in dose and concentration-dependent serious therapeutic failures and/or serious adverse drug reactions), as well as for drugs with non-linear pharmacokinetics due to limited solubility and solid dispersion formulations.

Australia, Canada, Colombia, the EU, Japan, New Zealand, Singapore, South Africa, Switzerland, and the WHO require both fasted and fed studies for products with specific formulation characteristics and complex technology involved (e.g., microemulsions, nanoparticles, solid dispersions) when the products can be administered either the fasting or fed state. Similarly, both fasted and fed studies are required for products where it is known that the innovator company evaluated different manufacturing technologies during the pharmaceutical development, and these formulations exhibited different food effects, even if the selected technology for the to-be-marketed product is not complex (e.g., micronization). However, Argentina, Mexico, Republic of Korea, and Chinese Taipei do not require both fasting and fed studies for an IR dosage form with specific formulation characteristics or for complex formulations where a difference in food effect is likely.

As the pre-dose meal may affect the absorption of the drug, it is important that the meal given to the study participants is standardized and the intake schedule is strictly controlled for all survey participants. Unless a different type of meal or diet is indicated is specified in the dosing instructions for the comparator product, a high-fat, high-calorie meal is generally used to ensure maximal perturbation of systemic bioavailability of the drug from the drug product. The caloric content of the meal and the distribution of the calories between carbohydrates, fat, and proteins are defined in the guidance documents of Argentina, Australia, Canada, the EU, Japan, Mexico, New Zealand, Republic of Korea, Singapore, South Africa, Switzerland, the United States and the WHO, but not specified in the guidance documents of Brazil, Israel, Colombia, and Chinese Taipei.

### Selection of subjects

For all survey participants, it is expected that BE studies should be conducted using healthy subjects to minimize the risk to study subjects and to reduce the inter- and intra-subject variability not attributable to the medicinal product itself. Additionally, healthy subjects are easier to recruit and generally do not require specialized medical care. There are circumstances where the safety profile of the drug precludes the administration to healthy subjects; as a result, a BE study in patients may be necessary. The responsibility to approve the proposed conduct of a BE study including the ethical acceptability of using healthy subjects largely falls on the research ethics board. It is important to note that the use of patients generally implies a study at steady state.

As described in the section **Single Dose and Multiple Dose Studies**, when the highest strength is not tolerated in healthy subjects, some jurisdictions may prefer conducting the BE study using an intermediate strength in healthy subjects rather than a study in patients using the highest strength. As discussed previously, if a biowaiver for additional strengths are requested, the strength to be used in the study may vary among the BEWGG members [29].

In a BE study where the product is intended for the treatment of only one gender (e.g., oral contraceptives), Australia, Brazil, Israel, Japan, Chinese Taipei, and the United States recommend that the studies be conducted in subjects of the same gender for which the drug product is intended. For Canada, the EU, Mexico, New Zealand, Republic of Korea, Singapore, South Africa, and Switzerland, studies conducted with the relevant gender is not mandatory; however, Mexico and Saudi Arabia will require a justification in the study protocol with respect to pharmacokinetic differences, ethical aspects, etc., if a specific gender is selected. Argentina, Colombia, and the WHO do not address this aspect in their guidance documents.

For a product intended for the treatment of the general population (e.g., antibiotics), the BE studies should be conducted with subjects of both sexes for Mexico, Chinese Taipei, and the United States. For Israel, studies with subjects of only one gender would be acceptable in cases where the effect of bioavailability is not gender specific. Argentina, Australia, Canada, Colombia, the EU, Japan, Republic of Korea, New Zealand, Saudi Arabia, Singapore, South Africa, Switzerland, including Brazil, and the WHO would accept studies conducted with subjects of the same sex even when there may be differences in bioavailability between sexes since the same formulation difference is expected in both sexes. As a result, the same test/comparator ratio is expected.

In the case of endogenous substances, where it is already present in the human body, conducting the BE study in a sex with lower levels of the endogenous substance would decrease the impact of the endogenous substances on the formulation effect being evaluated in the BE study. Although not defined in all relevant guidance documents, this approach would generally be considered appropriate by all survey participants.

Conducting a BE study in a population from a different region or with a demographic representation of the intended local market would be acceptable in Argentina, Australia, Brazil, Canada, Colombia, the EU, Israel, Japan, New Zealand, Saudi Arabia, Singapore, South Africa, Switzerland, Chinese Taipei, the United States, and the WHO. However, the Republic of Korea has specified that BE studies should be conducted using the local population.

Some products are intended for the treatment of a disease state in a specific age group. For example, products for dementia are usually administered to treat elderly people. Argentina, Australia, Brazil, Canada, Colombia, the EU, Japan, Mexico, New Zealand, Republic of Korea, Singapore, South Africa, Switzerland, Chinese Taipei, and the WHO, do not require studies in the indicated age group, while the Saudi Arabia, Israel, and the US recommend the study design to include as many subjects as possible from the intended age group.

During the conduct of a BE study, adverse events such as nausea, vomiting or hypotension may occur. When stated in the study protocol, Argentina, Australia, Brazil, Canada, Colombia, the EU, Israel, Mexico, New Zealand, Republic of Korea, Singapore, South Africa, Switzerland, Chinese Taipei, the United States, and the WHO, accept the administration of concomitant medicines to prevent predicted adverse events. For instance, when BE is tested between opioid formulations, naloxone is concomitantly administered to prevent adverse events such as nausea and vomiting. Japan and Saudi Arabia assess the situation on a case-by-case basis, but also require that administration of the concomitant medication is stated *a priori* in the study protocol.

During the recruitment of volunteers to be enrolled in a BE study, to minimize the variables which could interfere with the formulation effect, medical examination of potential subjects is necessary to ensure that the subjects are healthy and comply with the specific study protocol requirements. Argentina, Australia, Canada, Colombia, the EU, Israel, Japan, Mexico, New Zealand, Republic of Korea, Singapore, South Africa, Switzerland, Chinese Taipei, the United States, and the WHO, state that the medical examination is determined by the protocol and should include the medical history and results of routine tests of liver, kidney and hematological functions including specific parameters regarding the drug, while a minimum set of medical investigations is mandatory regardless the content of the protocol for Brazil. The US has also published some (draft) product specific guidance documents describing additional medical examination recommendations for studies involving certain drugs, such as cobicistat, divalproex sodium, lapatinib ditosylate, baricitinib, and valsartan [36–40].

Although the investigator should strictly follow the study protocol and its inclusion and exclusion criteria, the results of medical laboratory examination of the volunteers may not necessarily meet established normal ranges. Where the results of the laboratory examinations do not comply with established normal values, in Argentina, Australia, Canada, the EU, Mexico, New Zealand, Saudi Arabia, Singapore, South Africa, Switzerland, and Chinese Taipei, the clinical significance is assessed by the principal investigator and/or additional medical opinion. For the United States and the WHO, these subjects should not be included, except in rare cases. Brazil, Colombia, Israel, Japan, and Republic of Korea assess the situation on a case-by-case basis.

### Analyte to be measured

The BE study investigates the pharmaceutical perspective of drug release from the dosage form, as well as the clinical perspective of assessing whether the pharmacokinetic profile of the active moiety/drug is sufficiently similar between the test and comparator products. Consequently, it is necessary to measure the quantity of parent drug available in the biological matrix over time, even if inactive, if we want to compare the release from the dosage form.

All the regulators recommend that the parent drug should be measured, whenever possible, as it is considered the most sensitive entity to detect formulation differences between the study products. The metabolite, on the other hand, is the result of the drug metabolism following the release and absorption (i.e., the formulation effect). In those cases where the parent drug is inactive and plasma levels are highly variable or very low due to its rapid metabolism, it is acceptable to measure the active metabolite. The use of the metabolite data *in lieu* of the active parent drug data may also be considered acceptable if the parent drug could not be precisely and accurately quantified (e.g., due to very low levels). This approach would be considered acceptable when the metabolite is reflective of the extent of absorption of the parent (i.e., not formed due to a saturable process). However, the use of a metabolite as a surrogate for the active parent compound is typically only accepted under exceptional cases, as recent advancements in bioanalytical techniques have made accurate and precise measurement of the parent drug uncommon. In instances where the parent drug cannot be measured reliably, the BE criteria will be applied to the primary metabolite data, and the applicant should provide data to support the inability to measure the parent drug accurately.

Notably, for Argentina, Australia, the EU, New Zealand, Saudi Arabia, Singapore, South Africa, and Switzerland, the metabolite may be considered as the primary analyte for BE determination in situations where it is known that the metabolite is the most sensitive. For example, this occurs when the metabolite is rapidly absorbed as seen with ezetimibe, where the sum of unchanged drug and its metabolites must be considered. On the other hand, Israel, Chinese Taipei, and the United States, recommend the metabolite to be quantified as supportive information if metabolites are formed substantially through pre-systemic metabolism and contribute significantly to the safety and/or efficacy of the product.

In the case of the Chinese Taipei, the BE determination is based on the data of the parent drug. However, if the metabolite data do not fulfill the BE criteria, its potential impact on patient safety and efficacy would be considered when making the regulatory decision.

For the United States, the regulatory decision is based on the 90% CI approach from the parent drug, although the applicant should present as supportive evidence the primary metabolite when it is formed directly through pre-systemic metabolism (gut wall or lumen) and it contributes to the safety and efficacy of the product. The United States also recommends collecting PK data on both parent and metabolite for pro-drugs; however, in both cases, BE criteria will only be applied to the parent data and the metabolite data will be used as supportive evidence. If the parent drug concentrations are too low to allow reliable analytical measurement in blood, plasma, or serum for an adequate length of time, then the metabolite data obtained from these studies should be subject to the 90% CI approach for BE demonstration.

Japan treats this issue on a case-by-case basis based on rapid metabolism, feasibility of quantitative measurements, and safety and efficacy of the drug product. However, if both the parent and metabolite have pharmacological activity, Japan then generally recommends that both analytes should be used to determine the BE based on the 90% CI evaluations, even if the metabolite is systematically formed.

### Endogenous substances

It is necessary to consider the initial presence of the analyte in subjects prior to drug administration in order to accurately assess the quantity available from study drug products. All survey participants recommend baseline correction in such cases.

Argentina, Brazil, Canada, Colombia, Mexico, Singapore, Switzerland, and the United States accept the measurement of three time points before dosing to set a baseline of the endogenous substance for subtraction from the average concentrations found from the samples collected after dosing. Australia, the EU, New Zealand, Republic of Korea, Saudi Arabia, South Africa, and the WHO also allow this procedure for substances without (or with small) daily variations. For those with daily variations, a curve for the endogenous compound should be generated and the result for the appropriate time subtracted. The baseline correction method should be described and justified in the protocol. Israel, Japan, and Chinese Taipei assess the methodology of performing this correction on a case-by-case basis.

### BE criteria

BE is determined by comparing the primary pharmacokinetic parameters obtained from each of the formulations, that reflect the rate and extent of absorption. The pharmacokinetic parameters are generally linked to the amount of drug present in the tested formulations as such, all survey participants recommend that the measured drug content of the biostudy test and comparator product batches be within 5% of each other. In the instances where a 5% maximum difference cannot be achieved, Canada, Colombia, the EU, New Zealand, Saudi Arabia, Singapore, South Africa, Switzerland, and the WHO accept a potency correction if this is pre-defined and justified in the protocol. Additionally, Canada also requires that the analysis of potency documented in the certificates of analysis is conducted within 6 months prior to the start of the study.

The primary parameters used to demonstrate BE commonly include the area under the blood concentration versus time curve (AUC), indicating the extent of absorption/exposure, and the maximum blood concentration (C_max_), which is related to the rate of absorption/maximum exposure and drug safety. Depending on the jurisdiction, the determination of BE may involve the use of either AUC to the last quantifiable concentration (AUC_0-t_) alone or both AUC_0-t_ and AUC extrapolated to infinity (AUC_0-∞_). The AUC_0-t_ parameter is calculated using a non-compartmental trapezoidal rule (linear or linear-log) from time zero to time “t”, where “t” is the time of the last quantifiable drug concentration determined experimentally. While the AUC_0-∞_ parameter is calculated in a similar manner with the exception that the area under the curve from the last quantifiable concentration to infinity is extrapolated using the terminal disposition rate constant (k_el_) as C_t_/k_el_, where C_t_ is the last measurable concentration.

Argentina, Australia, Brazil, Canada, Colombia, the EU, Israel, Japan, Mexico, New Zealand, Republic of Korea, Saudi Arabia, Singapore, South Africa, Switzerland, and the WHO require AUC_0-t_ as the relevant AUC parameter to conclude BE. Whereas the Chinese Taipei and the US additionally recommend AUC_0-∞_.

The 90% confidence interval for AUC_0-t_ (or AUC_0-t_ and AUC_0-∞_) should be within acceptance ranges of 80%–125% for Argentina, 80.0%–125.0% for Canada and 80.00%–125.00% for the remaining survey participants. For Argentina, the confidence interval of the relative mean C_max_ of the test to comparator product should be within 80%–125%, while for the other participants it should be within 80.00%–125.00% to determine BE. However, Canada does not require a 90% confidence interval for C_max_, instead, it requires the relative mean C_max_ of the test to comparator product (i.e., point estimate) should be within 80.0%–125.0%.

**FIGURE 1:**
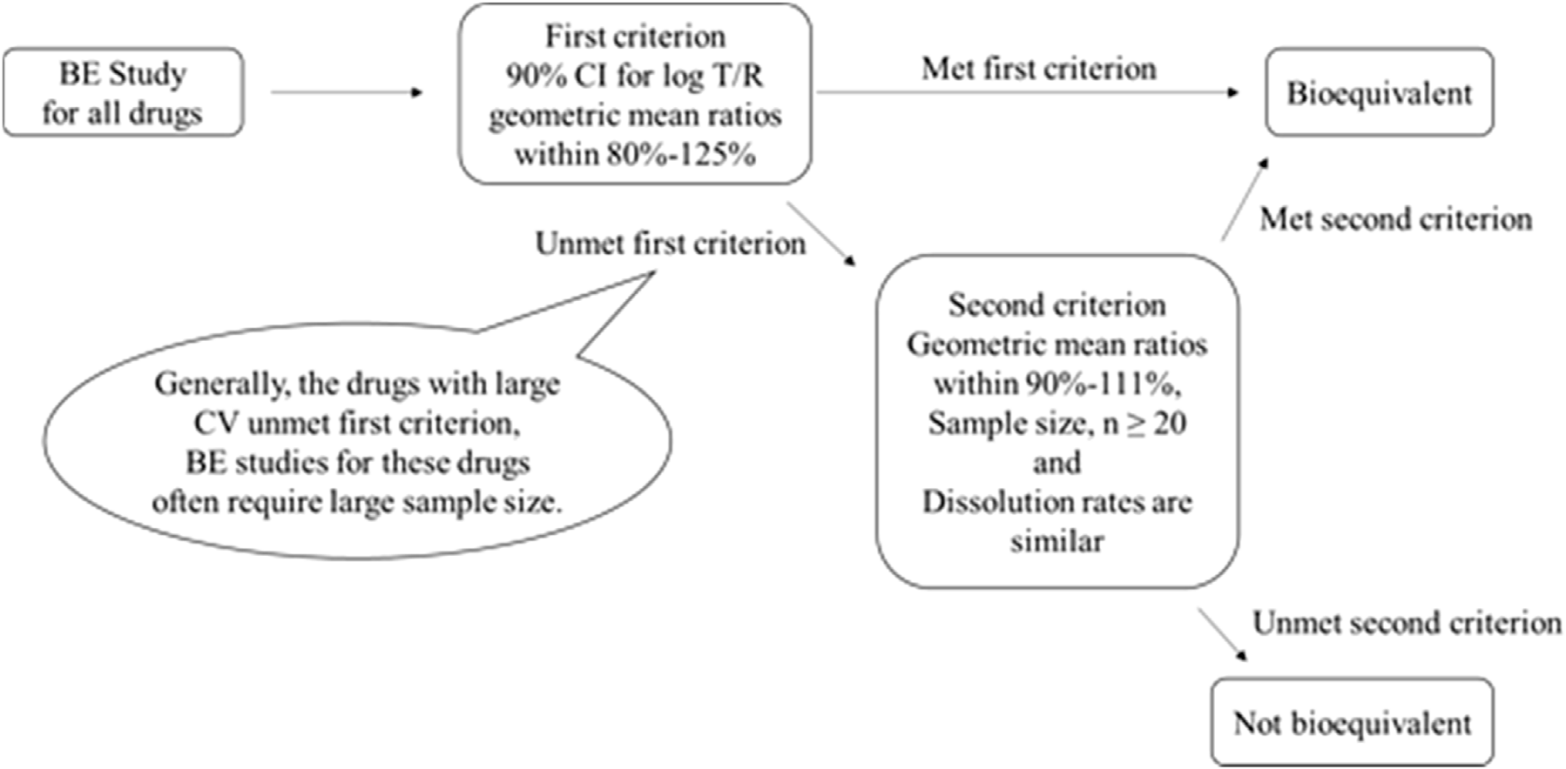
Criteria adopted by Japan for concluding the BE of the biostudy products.

Whereas Japan concludes BE by also considering the number of study participants and the dissolution rates of the test and comparator products if the confidence intervals for AUC_0-t_ and C_max_ do not fall within the acceptance range of 80.00%–125.00%. The second criterion, requires that the geometric mean ratio for C_max_ and AUC_0-t_ should be between 90.00% and 111.11% when there are 20 or more participants. The dissolution rates of the products compared should also be similar in multiple dissolution media. However, the second criterion cannot be applied if the number of participants is below 20 subjects or if the dissolution rates of the products are not similar, as shown in the Figure 1.

When conducting multiple dose studies for IR formulations, Argentina, Australia, Brazil, Colombia, the EU, Israel, Japan, Mexico, New Zealand, Republic of Korea, Saudi Arabia, Singapore, South Africa, Switzerland, Chinese Taipei, the United States, and the WHO, use the area under the concentration versus time curve at steady state over the dosing interval (AUC0-tau) and C_max_ at steady state (C_max,ss_) for BE determination. The 90% confidence interval of AUC0-tau and C_max,ss_ for the ratio test/comparator should be within 80.00%–125.00% inclusive. Except for Argentina where the acceptance range is 80%–125%. For IR products, the shape of the descending part of the concentration–time curve depends only on the elimination rate (ke) of the drug and not on the absorption rate (ka) from the dosage form. Therefore, Ctau, which is used to assess the impact of the absorption rate (ka) on the shape of the curve in prolonged release product, does not need to be compared for IR product in these survey participants. However, in drugs with flip-flop kinetics (absorption rate limited elimination) where ka<ke, the curve shape depends on the drug product. In Canada, the determination of BE is based on AUC0-tau, Cmax, ss and the minimum concentration at steady state (C_min,ss_). The 90% confidence interval for AUC0-tau should be between 80.0% and 125.0% inclusive, while the relative mean of C_max,ss_ should be within 80.0%–125.0% inclusive and the relative mean Cmin,ss should be not less than 80.0% inclusive.

For drugs known to exhibit high variability [within-subject coefficient of variation (CV) > 30%], some survey participants may allow the widening of the acceptance range of the 90% confidence intervals based on the CV of the comparator product. It is important to note that the variability of the comparator must be obtained in the BE study where the acceptance range is being widened) by dosing replicates of the comparator product, rather than relying on data from the literature or a previous study. Argentina, Australia, Brazil, Colombia, the EU, Mexico, New Zealand, Republic of Korea, Saudi Arabia, Singapore, South Africa, Switzerland, and Chinese Taipei may permit the widening of the acceptance range of the confidence interval for only C_max_, if it is shown that these larger C_max_ differences have no clinical relevance. On the other hand, the US accepts the scaled average BE for both C_max_ and AUC, while Israel widens the acceptance range for both C_max_ and AUC. The WHO allows the confidence interval to be widened for C_max_, and the Prequalification of Medicines Programme may also accept it for AUC. Additionally, as mentioned previously, Japan has a second criterion based on assessing the points estimates, with additional criteria for a sample size larger than 20 subjects and similar dissolution profiles in multiple dissolution media. Whereas Canada accepts the widening of the acceptance range of AUC, since the acceptance standard for C_max_ is based on the point estimate only.

**FIGURE 2:**
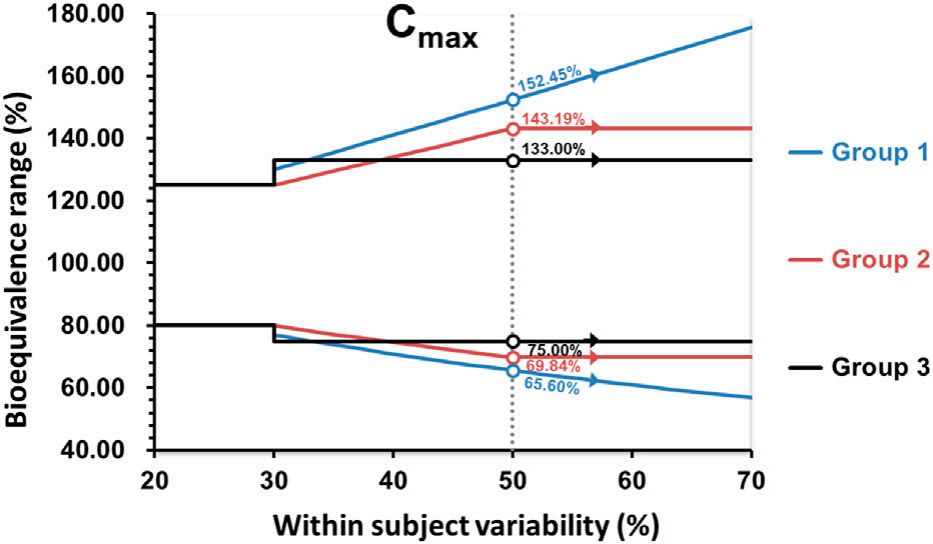
Comparison of the BE acceptance range for C_max_. Group 1: United States; Group 2: Argentina, Australia, Brazil, Colombia, the EU, New Zealand, Republic of Korea, Saudi Arabia, Singapore, South Africa, Switzerland, WHO; Group 3: Israel, Mexico, and Chinese Taipei.

**FIGURE 3:**
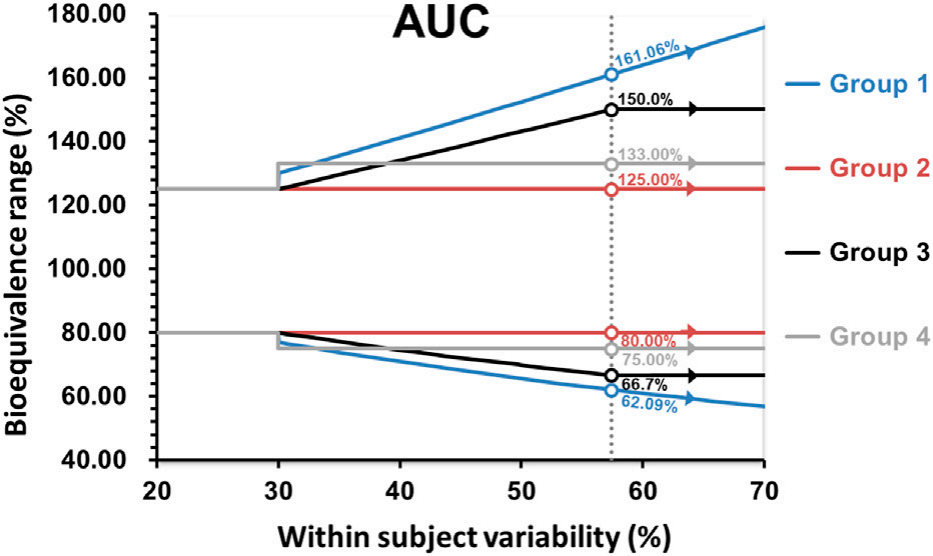
Comparison of the BE acceptance range for AUC. Group 1: United States; Group 2: Argentina, Australia, Brazil, Colombia, the EU, Mexico, New Zealand, Republic of Korea, Saudi Arabia, Singapore, South Africa, Switzerland, Chinese Taipei and WHO; Group 3: Canada; Group 4: Israel.

It is important to note that for Argentina, Australia, Brazil, Colombia, the EU, New Zealand, Republic of Korea, Saudi Arabia, Singapore, South Africa, Switzerland, and the WHO, the maximum widening of the acceptance range is 69.84%–143.19% for drugs exhibiting CV ≥ 50% or more. Whereas for the United States, the scaled average BE is used without any limit, potentially allowing the acceptance range for the 90% confidence interval to be widened beyond the above described limits. The widening applied in the first group is based on a proportionality constant of 0.76, calculated as ln(1.25)/0.294, where 0.294 is the regulatory limit σ _w 0_ that corresponds to CV = 30% and the minimum value of CV where the widening is applied at CV > 30%. The proportionality constant that would correspond to the methodology employed by the US-FDA is 0.892. In the United States, scaling is conducted when the CV > 30% (i.e., s_WR_ = 0.294), but with σ_w0_ = 0.25 (CV of 25.4%) as described by the Food and Drug Administration Draft Guidance on BE Studies With Pharmacokinetic Endpoints for Drugs Submitted Under an Abbreviated New Drug Application [41]. In Canada, the widening of the acceptance range of the 90% CI is applied to AUC_0-t_, using the 0.76 proportionality constant as defined in the first group of survey participants. The expansion of the BE acceptance interval may be permitted up to a maximum width of 66.7%–150.0% (equivalent to a scaled criterion for CV = 57.4%). The requirement for C_max_ remains the same given that the parameter does not involve the use of a 90% confidence interval. In Chinese Taipei and Mexico, if pre-specified in the study protocol, as long as CV % ≥ 30%, the acceptance range of the confidence interval for C_max_ can be scaled to a fixed range directly (i.e., 75.00%–133.00%), but cannot be expanded further. In Israel, the widening of the acceptance range of the 90% confidence interval is applied to AUC_0-t_ and C_max_, up to a maximum width of 75.00%–133.00% for drugs exhibiting CV ≥ 30%. The summary of how members wide the confidence interval for Cmax and AUC are shown in the Figures 2, 3.

For drugs with a narrow therapeutic index or considered critical dose drugs in Canada, the acceptance range for the 90% confidence intervals for AUC are narrowed from 80.00% to 125.00% to 90.00%–111.11% in Australia, Colombia, the EU, Israel, New Zealand, Saudi Arabia, Singapore, South Africa, Switzerland, and the WHO, and to 90.0%–112.0% for Canada. The 90% confidence interval for C_max_ is narrowed on a case-by-case basis, depending on the clinical relevance of C_max_, for the same countries except for Canada. For example, in the EU, the acceptance range of C_max_ for cyclosporine is narrowed, but that of tacrolimus is not. In Canada, the 90% confidence interval for C_max_ should be within 80.0%–125.0%, inclusive.

In the United States, studies for drugs with a narrow therapeutic index should use a fully replicated, 4-way cross-over design to scale the BE limit to the variability of the comparator product. They should also simultaneously compare the mean and within-subject variability of the test and comparator products as presented by the Draft Guidance on BE Studies With Pharmacokinetic Endpoints for Drugs Submitted Under an Abbreviated New Drug Application. The test product should pass the scaled and unscaled limits of 80.00%–125.00% [41].

In Mexico, a drug is considered to have a narrow therapeutic index when the ratio between the median lethal dose (DL50) and the median effective dose (DE50) is less than 2. Critical dose drugs are defined as drugs where small differences in dose or concentration in the body lead to serious therapeutic failures or serious adverse reactions. Thus, for drugs with a narrow therapeutic index, the 90% CIs for AUC should be within 90.00%–111.11% and for C_max_ the 90% CIs should be within 80.00%–125.00%. While for critical dose drugs, the 90% CIs for both AUC and C_max_ should be within 80.00%–125.00%.

Argentina, Brazil, Japan, Republic of Korea, and Chinese Taipei have not defined specific acceptance ranges for studies conducted with products containing drugs with a narrow therapeutic index.

### Other aspects considered in BE studies

#### Long half-life drugs

For long half-life drugs, all the survey participants agreed that for IR products, the sample collection schedule may be stopped at 72 h to generate a truncated AUC (i.e., AUC_0-72_). This is with the assumption that absorption will be complete in most subjects within 72 h. As a result, Argentina, Australia, Brazil, Canada, Colombia, the EU, Israel, Japan, Mexico, New Zealand, Republic of Korea, Saudi Arabia, Singapore, South Africa, Switzerland, Chinese Taipei, and the WHO would accept a sample collection schedule of at least 72 h. However, the US will allow the truncation to occur at a time shorter than 72 h. In this case, it is necessary that the truncation time encompasses the complete absorption phase.

In a hypothetical scenario where the study is truncated at 72 h and some samples at 72 h were not collected, South Africa, and the US would consider the “t” (as in AUC_0-t_) as the time when the last sample collection occurred. Australia would expect extrapolation for the final time point (or interpolation if a longer time was sampled). Canada and the WHO would only consider the subject for C_max_ calculation and not for AUC_0-72_ determination. Argentina, Brazil, Colombia, the EU, Israel, Japan, Mexico, New Zealand, Republic of Korea, Saudi Arabia, Singapore, Switzerland, and Chinese Taipei would handle this issue on a case-by-case basis.

#### Partial AUC

For drugs where the onset of action is crucial for therapeutic effect (e.g., analgesic for rapid pain relief), some regulators may have additional parameters to conclude BE. Some survey participants consider that partial AUCs are important as they could be closely linked to the onset of the clinical effect of the formulation. Thus, Canada and the US reported that partial AUC assessment might be necessary. On the other hand, Argentina, Australia, Brazil, Colombia, the EU, Japan, Israel, Mexico, New Zealand, Republic of Korea, Saudi Arabia, Singapore, South Africa, Switzerland, Chinese Taipei, and the WHO do not have requirements for partial AUC for IR products. Additionally, Colombia, the EU, Republic of Korea, South Africa, Switzerland, and the WHO stated that if differences in the time required for the manifestation of the drug effect could affect its clinical usefulness, then T_max_ is used as a parameter for evaluation of BE.

In the EU, the median and range of T_max_ are assessed visually. Although a specific criterion has not been defined, it is expected that T_max_ occurs in the same sampling time for test and comparator, or in adjacent sampling times (or in sampling times that are considered similar) since a slight imbalance may cause the median value to change between two adjacent sampling times. For example, if T_max_ in one product is at 30 min in 10 subjects and at 45 min in 9 subjects, the median is 30 min, and if in the other product T_max_ occurs at 30 min in 9 subjects and at 45 min in 10 subjects, the median is 45 min. Therefore, a difference in a single subject between two adjacent points may change the median value.

Canada requires an additional parameter such that the relative mean area under the curve to the time of the maximum concentration of the comparator product (AUCReft_max_) of the test to comparator formulation should be within 80.0%–125.0% inclusive. The AUCReft_max_ ratio for each subject should be calculated using values for test and comparator products obtained with that subject, and not using a central value (mean or median) for the comparator product. It is worth noting the US may truncate and use a partial AUC as an extra metric for BE determination as well.

For those regulators whose studies should be done using a partial AUC, Japan and the US have defined the 90% confidence interval to be from 80.00% to 125.00%. The US also indicated the possibility to perform a scaled BE calculation for partial AUC in the case of highly variable drugs (CV > 30%).

#### Outliers

During BE studies, it is possible to encounter plasma concentration values that deviate significantly from the rest of the study data. While the exclusion of outlier data based on the other data in the same study may seem appropriate, such exclusion is not recommended as it can be interpreted as an attempt to modify the dataset to reach the study objective, which is to demonstrate BE.

Exclusion of aberrant data from subjects after the completion of the study is not permitted by Argentina, Australia, Brazil, Colombia, the EU, Japan, New Zealand, Republic of Korea, Saudi Arabia, Singapore, South Africa, Switzerland, Chinese Taipei, the United States, and the WHO. However, Canada would consider outliers based on statistical evaluation. In Canada, the justification for excluding outliers from a study is to be supported by a simple outlier test, such as the studentized residual being greater than 3. Additionally, the outlier should fall outside the range of all values for AUC and C_max_ regardless of the formulation. The Canadian guideline outlines that no more than 5% of the subjects can be considered outliers and excluded from the study, unless there are 20 or fewer subjects, in which case only one subject may be removed. Israel has not addressed this issue in their guidance documents.

In Mexico, eliminating aberrant data based solely only on a statistical evaluation is not permitted. Instead, it is necessary to investigate the possible causes behind the appearance of one or more extreme values requiring scientific evidence to justify the removal of outliers. Australia, Brazil, Colombia, the EU, New Zealand, the Republic of Korea, Saudi Arabia, Singapore, Switzerland, Chinese Taipei, the United States, and the WHO specify that exclusion of data for statistical or pharmacokinetic reasons alone is not acceptable. However, if the reason for the outlier result were identified (e.g., vomiting, diarrhea occurring shortly after administration of study medication), and if the reasons for exclusion and the procedure for its identification were described in the study protocol and followed, its exclusion would be acceptable.

Re-analyzing anomalous concentrations may be feasible to confirm if they are caused by cross-contamination or carry-over from a previous administration, but this is only acceptable for pre-dose samples with measurable concentrations. However, the re-analysis of a sample with an unreliable concentration due to discrepancies with the pharmacokinetic profile (i.e., re-analysis due to pharmacokinetic reasons) is not acceptable in Argentina, Australia, Brazil, Colombia, Canada, the EU, Japan, Mexico, New Zealand, Republic of Korea, Saudi Arabia, Singapore, South Africa, Switzerland, Chinese Taipei, the United States, and the WHO. Israel would treat this issue on a case-by-case basis.

As previously described, in replicate design studies for highly variable drugs, the acceptance range can be widened, or the average BE acceptance criteria can be scaled, based on the observed intra-subject variability of the comparator product. The identification of outliers in such studies become important as they may inflate the variability estimation and confidence interval ranges. The methodology to identify outliers for replicate design studies is not described in current guidelines of the IPRP BEWGG members. However, in the EU, Singapore, South Africa, Chinese Taipei, and the WHO, it is expected that the influence of outliers be assessed by sensitivity analyses showing the results including and excluding the data that might be considered as outliers [42].

## Discussion

BE studies are the main tool for comparing the bioavailability of two medicinal products containing the same active substance, and thus establishing their interchangeability. The recommendations for conducting these studies among members of the IPRP BEWGG for IR solid oral dosage forms are similar in certain aspects (e.g., single/multiple dose and sample size) but they significantly differ in other aspects (e.g., fasting/fed condition and pAUC.

### Fed vs. fasted state

Demonstrating BE with respect to food intake presents a complex issue. The US and Japan have relatively standard approaches. While, the other survey participants, including the EU, define these requirements on a case-by-case basis to minimize the number of studies where possible. As stated earlier, these participants refer to the dosing condition stipulated in the package insert of the comparator product to determine whether a fasting or fed study is recommended. However, exceptions to this rule are made if the recommended meal condition for the comparator product is based on tolerability concerns only (assuming this is drug-dependent and not formulation-dependent), or where there are complex formulation effects which may affect drug bioavailability. This latter scenario is often more challenging for regulators and industry due to confidentiality issues concerning the innovator formulation, e.g., patented manufacturing technologies (e.g., micro-emulsions) or specific formulation characteristics (e.g., micronization). Regulators may request fed studies as showing BE in the fasting state, yet it may not guarantee BE in the fed state. However, the reason for this recommendation may not be apparent to generic companies when there appears to be no food effect for the comparator product. Some agencies address this issue with product-specific guidances. For instance, the European label specifies that the comparator products for sirolimus and everolimus (tablets—either intact or as a suspension, or dispersible tablets) should be consistently taken with or without food. Given that the specific formulation (e.g., particle size and excipients) is known to be critical to the performance of the formulation under fed conditions, it cannot be assumed that the effect of food will be the same regardless of formulation [33, 34]. Additionally, both fasting and fed BE studies are required for lapatinib, because, according to the label of the comparator product, lapatinib should be administered in a standardised manner at least 1 h before food or at least 1 h after food. There is a notable difference in absorption (2-3-fold difference in AUC) when lapatinib is administered 1 h before vs. 1 h after a meal. Due to the strict requirement regarding standardization of dosing for individual patients, demonstrating BE needs to be shown under both (semi-) fasting and semi-fed (1 h after food) conditions, as outlined in the EMA Lapatinib film-coated tablet 250 mg product-specific BE guidance [35]. Additional complex examples include rivaroxaban and dasatinib [4, 43]. The need for an additional study may be waived if the test product uses the same technology and similar excipients as the comparator product. This is provided that generic manufacturers have sufficient information on these aspects. For instance, in the case of tadalafil, it may be possible to waive the BE study in the fed state if it can be shown that the excipient composition and manufacturing method is similar to those of the comparator product.

There is a potential misconception regarding recommending both fasting and fed studies only in the presence of evidence of a product-dependent food effect with the comparator product. Information on product-dependent food effects is not widely investigated, thus the lack of evidence demonstrating a food effect with the comparator product does not necessarily indicate that there is an absence of a food effect with the proposed test product. BE conducted under fed conditions is not recommended or may be waived by most survey participants. A harmonized recommendation to conduct BE studies under fed conditions would provide sufficient data to elucidate whether the food effect is not only active substance-dependent but also product-dependent.

### Highly variable drugs

There is significant differences in regards to the assessment of highly variable drugs. The differences are in the scaling parameters and the statistical methods. In the United States, the average BE is scaled with no fixed limits for AUC and C_max_. Whereas the other survey participants allow the acceptance limits to be widened utilizing a similar approach where widening of the acceptance ranges for AUC and/or C_max_ are based on the within-subject CV of the comparator and have a limit. There are some exceptions in Israel, Mexico, and Chinese Taipei for Cmax, and Israel for AUC, where the acceptance range is widened to a fixed value. Using the scaled average BE might be considered statistically appropriate because the limits do not become a random variable, whereas widening the limits might be considered more straightforward and easier to understand. A few members still use a widened, but fixed, acceptance range for all highly variable drugs in the same way. These differences have complicated the harmonization in this field. A similar situation is observed with the acceptance range of narrow therapeutic index or critical dose drugs, as the US scales down the average BE and compares intra-subject variability of the products, while the other survey participants use a fixed (narrower) acceptance range independent of the intra-subject variability.

### Rate of absorption where clinically relevant

The approaches used to assess equivalence in the rate of absorption for products with clinically relevant onset of action vary widely. The approach utilized by Canada, which relies on partial AUC until T_max_ of the comparator within each subject, is challenging, particularly because it cannot be applied to parallel designs. In addition, for replicate designs, it is not clear how to apply. This is due to the potential T_max_ occurrence of Tmax at two different sampling times in the periods when the comparator product is administered. In addition, the median of both periods could be a time where a sample has not been taken and it might not be estimable in those subjects where the levels of the test continue to be zero when the comparator product has reached C_max_. Most importantly, this partial AUCReft_max_ is highly variable and is assessed with a point estimate rather than 90% CI, raising methodological concerns, from a statistical standpoint. In the United States, in most cases the pAUC is truncated at a time with pharmacodynamic relevance. This can be difficult for the companies to define without being predefined in product-specific BE guidances. Additionally, it might not be estimable in those profiles where the levels of the test or the comparator levels remain at zero at the pre-defined cut-off point. Although these pAUCs can be assessed more easily than T_max_, which is a discontinuous variable, these pAUCs are much more variable than the usual primary pharmacokinetic parameters. Therefore, to demonstrate equivalence, a replicate design would be required to scale average BE or widen the acceptance range. The intra-subject variabilities are so large, that the widening up to a maximum of 50% CV would be of little value, since values >100% are frequently observed. Consequently, truncating the widening at CV = 50% would result in an extremely large sample size. The replicate design and the large sample sizes would complicate the development of these drugs, despite the process being simplified with the visual inspection of the median and range of T_max_. This methodology is highly questionable, as inferences should be based on confidence intervals. In addition, it seems that these pAUCs are recommended for very few active substances (e.g., triptans) by survey participants where pAUCs are assessed. In contrast, the visual comparisons of the median and range of T_max_ are required for a few more drugs (e.g., NSAIDs) by survey participants where T_max_ is used as the metric to ensure a similarly rapid onset of action. It can be concluded that if pAUCs were requested by survey participants that currently use T_max_ to compare the similarity in the onset of action, the marketed products that were authorized with T_max_ data would have been rejected due to the notably insufficient usual sample size determined by C_max_ intra-subject CV (%).

Certain countries or a minority of countries take unique approaches in several aspects, which are interesting for identifying opportunities for harmonization.

### Switchability

In Brazil, there is a concern that a larger variability in the test product could affect the switchability with the comparator product, hence the post-study power must be confirmed. This unique approach is surprising because the lack of power might be caused by other factors, such as a larger than expected formulation difference. Additionally, the failure to detect a difference in the intra-subject variabilities does not mean that they are similar as the null hypothesis is never proven. Moreover, if the test product exhibits lower variability than the comparator product, this difference may lead to the rejection of a product due to a larger than expected difference, even if it is within the BE range (e.g., 10% vs. the expected 5%). Furthermore, in the case of a parallel design, this approach is not applicable, as switchability based on intra-subject variability cannot be addressed with the variability obtained in a parallel design.

### Race

Hypothetically, a race may be more sensitive in detecting differences in a BE study if that race has a higher prevalence of extensive metabolizers. This reason for this is the shorter the half-life, the higher the sensitivity to detect differences in C_max_. However, the Republic of Korea’s requirement of recruiting local population seems to represent a different interpretation, as different races sometimes exhibit different pharmacokinetics. The difference would be detected in the ANOVA as a race effect, but that would be inconsequential for interchangeability because the same race effect would occur in the test and the comparator. The interchangeability would be affected in the case of a significant race-by-formulation interaction, which would be indicative of a different test/comparator ratio between races.

### Gastric pH

In Japan, if the *in vitro* dissolution data from the test and comparator products showed specific significant differences around pH 6.8 or between pH 3.0 and 6.8 for products containing basic drug products, subjects with low gastric acidity (achlorhydric subjects) should be employed in the BE study. This is because the percentage of Japanese people with low gastric acidity is larger than that in people of the EU and the United States. The first priority in Japan is to conduct a BE study enrolling subjects with low gastric acidity. It is also considered appropriate to conduct the study with healthy adult subjects by co-administration of gastric acid reducers (e.g., proton pump inhibitors). A parallel has been drawn with the requirements for studies with proton pump inhibitors in the EU, in addition to studies in the fasted state, for drugs with high solubility at acid pH but low solubility at more neutral pH. This aims to demonstrate that the products are bioequivalent not only in the acid conditions of the stomach but also in cases where the pH of the stomach is less acidic. This additional study has been required for those drugs where concomitant intake with proton pump inhibitors is frequent (e.g., dabigatran etexilate hard capsules) [44]. In the case of prasugrel, a study under increased stomach pH is required if the salt is changed or the base is used instead of the hydrochloride employed in the comparator product, due to different pH-dependent solubility of the salt and base. This increased pH can be achieved with a fed study or a study with proton pump inhibitor pre-treatment, such as lansoprazole 40 mg b.i.d. for 4 days [45], but a fed study is less specific in raising gastric pH as it also stimulates the secretion of bile.

### Parent vs. metabolite

While a consensus has been reached regarding the higher sensitivity of the parent in most active substances, it remains unclear under what scenario the active metabolite might be more sensitive to detect differences in the *in vivo* performance of products. This may explain why some survey participants recommend active metabolite data as supportive information, particularly when the metabolite is formed pre-systemically, although the consideration of metabolite data is a regulatory decision in the case of Japan and Chinese Taipei. It appears that the metabolite could be more sensitive in cases where the metabolite is formed in the intestinal lumen, or the enterocyte and its absorption is quicker than the parent (e.g., due to the different effect of efflux or uptake transporters). A good example is ezetimibe, known for enterohepatic recycling of the parent drug and more rapid absorption of the metabolite and thus, the sum of unchanged drug and its metabolites must be considered for BE [46, 47]. It would be necessary to identify the specific cases and reasons for the higher sensitivity of the metabolites to reach a systematic approach where active metabolite data should be factored in to decide on the BE of the products. This is particularly important considering that the current approach of requesting active metabolite data as supportive information is valued by the United States, unlike some of the other survey participants, if it is not going to be taken into account for regulatory-decision making.

### Long half-life

In the case of drugs with long half-lives, it is important to harmonize on an approach for situations where the sample is not available at 72-h. Imputation of that value may not be consistent with the use of model independent data, which is employed with trapezoidal rule for AUC estimation. Excluding these subjects can affect the statistical power of the study, which may be critical in multiple cases of missed 72-h samples. On the other hand, the combination of AUC_0-t_ in one period and AUC_0-72h_ in another period can artificially inflate intra-subject variability, particularly when the difference in AUC is large between adjacent truncation time-points, unlike in drugs with shorter half-lives. Another possibility would be to accept the sponsor’s pre-defined protocol, as such this decision could determine the success or failure of the study. Of course, truncation of AUC at times later than 72-h is acceptable, but it would be necessary to agree on whether earlier truncation (e.g., 48 h) could also be acceptable.

### Acceptance criteria

The conventional BE acceptance range varies between countries. In Canada and Israel, a single and no decimal unit are employed, respectively. It is important to note that in Canada, the assessment of C_max_ is based on the point estimate of that ratio, rather than the 90% CIs. It is also important to highlight that the probability of locating the true value in all the points of the CI remains the same. A similar approach is employed in Japan, where a BE study that does not meet the acceptance criteria based on the 90% CI can be approved based on the point estimate if the dissolution profiles are similar, the sample size is at least 20 and the point estimate is within ±10% (i.e., 90.00%–111.11%). It is interesting to note that BE demonstration with a pilot study of 12 subjects is not acceptable in Japan. However, a pivotal study with more than 20 subjects and the 90% CI outside the conventional 80.00%–125.00% acceptance range can be acceptable if the dissolution profiles, which may not be *in vivo* predictive especially for BCS class II/IV drug, are deemed to similar. Studies for products with normal variability (e.g., 26%) need 24 + 2 subjects assuming no difference between test and comparator. Consequently, a point estimate of 110.72% with a 90% CI from 98.0 to 125.1% would be approvable, even if this drug product does not have high variability.

### Adaptive designs

For the members who accept two-stage study designs, adaptive designs may or may not be included. In the case of non-adaptive designs, the sample size for both groups and the alpha expenditure (i.e., confidence level of the confidence intervals) in each stage are pre-defined. There are infinite ways to distribute the subjects and adjust the alpha expenditure. It is the sponsor’s responsibility to demonstrate that the consumer risk of reaching a false equivalence conclusion is not greater than 5% with the selected stage-sizes and consumer risk adjustment. In the case of adaptive designs, the sample size of the second stage is generally calculated based on the estimated variability of the first stage. Once again, it is necessary for the sponsor to demonstrate that the patient risk is not inflated above 5% globally. According to the WHO guidance, the sample size of the second stage could be “hypothetically” calculated not only using the observed variability of the first stage, but also with the observed point estimate of the first stage. However, in such a case it remains the responsibility of the sponsor to demonstrate that the consumer risk is not greater than 5% [22]. The WHO guideline only provides advice for the case of a non-adaptive design, where the sample size of both stages is predefined, and the sample sizes of both stages is the same, although not specifically specified except for the reference used in the guideline. According to Reference [48], if the sample size of both stages is the same, the confidence level does not need to be 95% in both stages, which corresponds to the conservative Bonferroni’s adjusted. Instead, it can be 94.12% (which corresponds to an alpha of 0.0294, since the corresponding confidence level is (100-(2·alpha·100)), as indicated in the EMA guideline [10]. However, that paper refers to parallel designs and it has been highlighted that the value is also unnecessarily conservative for cross-over designs [49]. A confidence level of 93.92% (alpha of 0.0304) could be used in the case of the Pocock design for 2 × 2 cross-over trials. The WHO guideline also includes the possibility of not spending any alpha in the interim analysis after stage 1. This is because BE is not intended to be demonstrated in the interim analysis (i.e., the confidence interval is not calculated). Instead, this information can be used for a futility analysis, which does not use any alpha, i.e., to stop the trial when demonstration of equivalence is unlikely with the pre-defined sample sizes. In Canada and the United States, the recommendation of method C published by Potvin et al is/was an acceptable approach [50]. However, additional papers [51, 52] provided different conclusions due to changes in the assumptions of the simulated scenarios. As a result, these simulations are not considered enough justification of preservation of the overall type I error (alpha) in the EU. In fact, as Maurer et al. explained none of the 4 variations investigated by Potvin et al. formally controls the type I error rate of falsely claiming ABE, even though the amount of inflation produced by Method C was considered acceptable. A major disadvantage of assessing type I error rate inflation using simulation is that without exploring all possible scenarios for the intended design and analysis, it is impossible to ensure control of the type I error rate = [5, 53].

### Outlier data

Most survey participants do not allow outlier data to be excluded. However, an investigation of the impact of outliers on the BE conclusion (i.e., a sensitivity analysis) is considered useful. This is because outlier values may also contribute to a false BE conclusion when the products are not actually bioequivalent. Additionally, allowing the exclusion of outlier values solely based on a statistical test (without an investigation), raises questions about validity.

### ICH M13A guideline

The survey results and discussions were shared with the ICH M13 Expert Working Group (EWG) and played an important role in identifying and analyzing gaps during the harmonization process. The draft ICH M13A guideline developed by the M13 EWG was endorsed by ICH on December 20, 2022, under *Step 2* [54].

The current draft of the ICH M13A guideline focuses on study design, principles for conducting BE studies, BE standards for IR solid dosage forms, and data analysis. The guideline also recommends that BE studies be conducted according to the principles and recommendations in ICH E6 guideline for Good Clinical Practice.

Furthermore, the ICH M13A draft guideline also describes studies involving multiple comparator products, studies with multiple test products, endogenous compounds, and other IR dosage forms (e.g., orally disintegrating tablets, chewable tablets, suspensions, fixed dose combination products) that are not addressed in the current review publication. However, the current review publication, along with previous publications [3, 29] from the IRPP BEWGG describe several topics that will be addressed under future ICH projects (e.g., ICH M13B and M13C guidelines). It is evident that the continued efforts of the IPRP BEWGG will have a notable impact with respect to promoting collaboration and achieving regulatory convergence and harmonization in the field of BE study performance.

## Conclusion

The survey results highlighted the commonalities and differences among the IPRP BEWGG participants concerning the overall design of BE studies and the acceptance criteria for demonstrating BE for IR solid oral dosage forms. All participants prefer the single dose cross-over design in healthy subjects with a sample size of 12 or more subjects and recommend the use of parent drug data instead of metabolite data whenever possible to establish BE. However, differences amongst participants mostly occurred in cases where fasting and/or fed studies are required, BE acceptance criteria for highly variable drugs and narrow therapeutic index drugs, and the use of partial AUCs when the rate of absorption is clinically relevant.

By comparing and contrasting the requirements of the different regulatory agencies and sharing the regulatory information with the ICH EWG for M13A [54], it is evident that the persistent efforts of the IPRP BEWGG are instrumental in identifying and supporting topics for ICH harmonization. As the ICH EWG focus shifts towards the drafting of the ICH M13B guideline (BE for Immediate Release Solid Oral Dosage Forms: Additional Strengths), the prior work of the IPRP BEWGG regarding additional strength biowaivers [3, 29] should benefit the ICH EWG.
